# What Lies Beneath: Sub-Articular Long Bone Shape Scaling in Eutherian Mammals and Saurischian Dinosaurs Suggests Different Locomotor Adaptations for Gigantism

**DOI:** 10.1371/journal.pone.0075216

**Published:** 2013-10-09

**Authors:** Matthew F. Bonnan, D. Ray Wilhite, Simon L. Masters, Adam M. Yates, Christine K. Gardner, Adam Aguiar

**Affiliations:** 1 Biology Program, The Richard Stockton College of New Jersey, Galloway, New Jersey, United States of America; 2 School of Veterinary Medicine, Auburn University, Alabama, United States of America; 3 Beaumont School, Cleveland Heights, Ohio, United States of America; 4 Museum of Central Australia, Araluen Cultural Precinct, Alice Springs, Australia; 5 Dept. Biological Sciences, Western Illinois University, Macomb, Illinois, United States of America; University of Pennsylvania, United States of America

## Abstract

Eutherian mammals and saurischian dinosaurs both evolved lineages of huge terrestrial herbivores. Although significantly more saurischian dinosaurs were giants than eutherians, the long bones of both taxa scale similarly and suggest that locomotion was dynamically similar. However, articular cartilage is thin in eutherian mammals but thick in saurischian dinosaurs, differences that could have contributed to, or limited, how frequently gigantism evolved. Therefore, we tested the hypothesis that sub-articular bone, which supports the articular cartilage, changes shape in different ways between terrestrial mammals and dinosaurs with increasing size. Our sample consisted of giant mammal and reptile taxa (i.e., elephants, rhinos, sauropods) plus erect and non-erect outgroups with thin and thick articular cartilage. Our results show that eutherian mammal sub-articular shape becomes narrow with well-defined surface features as size increases. In contrast, this region in saurischian dinosaurs expands and remains gently convex with increasing size. Similar trends were observed in non-erect outgroup taxa (monotremes, alligators), showing that the trends we report are posture-independent. These differences support our hypothesis that sub-articular shape scales differently between eutherian mammals and saurischian dinosaurs. Our results show that articular cartilage thickness and sub-articular shape are correlated. In mammals, joints become ever more congruent and thinner with increasing size, whereas archosaur joints remained both congruent and thick, especially in sauropods. We suggest that gigantism occurs less frequently in mammals, in part, because joints composed of thin articular cartilage can only become so congruent before stress cannot be effectively alleviated. In contrast, frequent gigantism in saurischian dinosaurs may be explained, in part, by joints with thick articular cartilage that can deform across large areas with increasing load.

## Introduction

Both eutherian mammals and saurischian dinosaurs evolved lineages of huge terrestrial herbivores. For eutherian mammals, both the Afrotherian and Laurasiatherian lineages gave rise to terrestrial giants, the proboscideans and the ceratotherian *Paraceratherium*, respectively [1]–[3]. In saurischian dinosaurs, the long-necked sauropods achieved body sizes one order of magnitude greater than the largest terrestrial eutherian mammals [4], [5]. Previous research indicates that a concatenation of metabolic, reproductive, climatic, and geographic factors influenced the circumstances and means by which gigantism was achieved in sauropods (e.g., [5]–[7]), proboscideans, and *Paraceratherium* (e.g., [2], [3]).

It has long been recognized that gigantism of the kind achieved frequently in saurischian dinosaurs occurred much more rarely among eutherian mammals [7], [8]. For example, many sauropod taxa easily exceeded 10,000 kg [7]–[9], whereas only a very small percentage of eutherian mammals ever reached these masses [7], [8]. In fact, the largest-known terrestrial mammal, *Paraceratherium*, achieved a body mass no greater than 20,000 kg, a value that is likely exaggerated from extrapolation [10], [11]. Therefore, given that the limb skeleton is an adaptable framework that moves and supports the body, long bone morphology would be predicted to show different scaling trends between eutherian mammals and saurischian dinosaurs related to their different frequencies of gigantism.

However, previous morphometric analyses have not revealed significant departures in dimensions and shape among large, columnar-limbed eutherian mammals (e.g., [12]) or sauropods [13]–[16]. Instead, once a columnar posture is achieved, other factors such as behaviors that limit excessive movements and reduce bending stresses come into play [17], [18]. In essence, very large vertebrates with columnar limbs reduce mobility to achieve stability and limit bone stress. Not surprisingly, isometric or negative allometric scaling of long bone dimensions and shape is common (e.g., [12]–[15], [19]), an outcome predicted by decreasing locomotor scope with increasing size. Often, such results are used to suggest sauropods and large eutherians had dynamically similar gaits and comparable athleticism (e.g., [16], [20], [21]) or that sauropods achieved efficient locomotion through stiff-legged stilt-like mechanics [14]. Most recently, Sander and colleagues [5] suggested that locomotor scaling effects could not adequately explain sauropod gigantism because they apply equally well to other vertebrates.

Whereas long bone dimensions and shapes provide a first order approximation for locomotor scope, such simplifications ignore a fundamental difference between eutherian mammal and non-avian dinosaur long bones: the formation (or lack thereof) of secondary centers of ossification in the chondroepiphysis. This difference has significant effects on the shape of the articular cartilage, its thickness, and how it is loaded. In eutherian mammals, the chondroepiphysis develops atop a well-organized, dense layer of sub-articular bone [22]. A secondary center of ossification (an epiphyseal bone) develops within the chondroepiphysis which eventually fuses distally with the endochondral bone front, leaving a thin layer of articular hyaline cartilage proximally above the now-ossified epiphysis [23]. In contrast, non-avian dinosaur long bones grew and developed like those of extant archosaurs: the chondroepiphysis develops above a less-organized, less-dense sub-articular bone front depressed into a collar of periosteal bone [23], [24]. In extant archosaurs, the articular cartilage is typically thick and consists of hyaline and fibrocartilaginous tissues [25], and portions of these regions can be associated with muscle insertion sites. No epiphyseal bones are known to have developed in non-avian dinosaurs, and a thick layer of articular cartilage formed above the calcified cartilage and sub-articular bone front [25], [26] as in extant archosaurs [27].

Regardless of the mechanism of how epiphyseal bone centers form, these differences in articular cartilage thickness and composition between archosaur and mammal long bones should result in distinct sub-articular shape scaling patterns between these clades. The combined region of calcified cartilage and bone underlying the articular cartilage is known as subchondral bone [28]. This region supports articular cartilage and acts in conjunction with this tissue as a shock absorber [28]–[30]. In this study, subchondral bone shape is represented as the proximal outline of the subchondral bone. Thus, changes in subchondral shape should reflect the adaptive role of subchondral bone in parlaying dynamic locomotor stresses absorbed through the articular cartilage. If there are scaling differences in subchondral shape between eutherian mammals and saurischian dinosaurs as predicted by their varied success as terrestrial giants, nowhere should these be most starkly revealed than in the largest taxa of each lineage. Ultimately, such differences in subchondral shape scaling have the potential to illuminate major trends in weight-bearing and locomotion not detectable using previous approaches. Therefore, we tested the hypothesis that the subchondral shape of humeri and femora in giant lineages of eutherian mammals and saurischian dinosaurs scaled differently with increasing size using 2-D geometric morphometrics (GM).

## Materials and Methods

### Specimens and Sample

For our study, we chose to test for shape changes in the subchondral surfaces in the humerus and femur because these elements are the most often compared bones in dinosaur and mammal studies. Moreover, these two elements both provide a majority of the mechanical support to their associated limbs, and the largest muscles that control locomotor movements of the entire limb insert or originate on these bones [14]. Additionally, humerus and femur length are highly correlated with body size in mammals, dinosaurs, and other taxa [31], [32], and are used here as a proxy for body size.

We examined 232 humeri and 224 femora across a range of archosaurian and mammalian taxa (Tables S1 and S2). Permission was obtained to examine specimens in the museums and institutions listed in Table 1, and data from previous studies was also utilized [14], [15], [26]. No permits were required for the described study, which complied with all relevant regulations. To ensure that our samples were comparable, we established several criteria. All specimens selected were adult or at least non-juvenile to avoid ontogenetic effects. A majority of the long bones examined for this study were museum specimens, but the bird and alligator long bones were acquired from skeletal material generated during a previous study on archosaur joint cartilage [26]. For our eutherian mammal sample, we focused on the clades Afrotheria and Laurasiatheria (containing the giant proboscideans and ceratotherians), whereas taxa from Saurischia (containing the giant sauropods) comprised our dinosaur sample. Giant fossil taxa in Afrotheria and Laurasiatheria have close living relatives (i.e., members of their immediate lineages are still extant). Although Ornithischia contains some giant taxa as well (e.g., *Shantungosaurus* [33]), we focused exclusively on Saurischia because, like our mammal sample, they possess close living relatives: Aves. We selected taxa within our lineages of interest that had upright, parasagittal hindlimbs (as well as forelimbs for quadrupeds), and avoided taxa with predominantly fossorial, volant, saltatory, or scansorial habits. For Afrotherians, we excluded taxa such as tenrecs and macroscelidids, but we included *Orycteropus* because, despite its burrowing habits, it is a terrestrial forager. The highly-derived limb morphologies of ungulates (e.g., functional incorporation of the ulna into the radius) have been shown to scale in unique ways that often effect the interpretation of allometric scaling patterns across larger mammal samples e.g., [34]. For this reason, ungulates were excluded from the Laurasiatherian sample.

**Table 1:** MANOVA of partial warp scores which collectively describe the shape of the humerus and femur in the samples.

Taxon	Element	n	Wilk’s λ	F-statistic	Df	p
Eutheria**+Monotremata	Humerus	132	0.000055	40.250	150.000, 237.000	<0.000001
Eutheria**+Monotremata	Femur	127	0.000381	20.380	144.000, 228.711	<0.000001
Saurischia*+*Alligator*	Humerus	100	0.007000	10.468	100.000, 96.000	<0.000001
Saurischia*+*Alligator*	Femur	97	0.014000	7.246	96.000, 94.000	<0.000001

In all cases, there are significant shape differences among the taxa.

** Eutheria was divided into several clades: Afrotheria, Perissodactyla (rhinos and tapirs only), and Felidae.

* Saurischia was divided into Sauropodomorpha and Theropoda (Aves inclusive).

To place our samples within a broad evolutionary and postural context, we examined additional outgroups within and outside our “giant” lineages. For clarity, we are using the term “outgroup” here to mean taxa successively distant from the giant taxa, not in a strict phylogenetic sense. For Afrotheria, *Hyrax* and *Orycteropus* comprised successive outgroup taxa to the giant proboscideans, whereas *Tapirus* and felid taxa formed successive Laurasiatherian outgroups for the ceratotherians. For Sauropoda, the sauropodomorphs *Plateosaurus* and *Massospondylus*, as well as the non-avian theropod *Allosaurus* and the avian theropod *Numida* were selected as successively distant outgroups. *Numida meleagris* was selected because of its predominantly terrestrial habits and because we had access to numerous wild (i.e., free range) individuals from a previous morphometric analysis [26]. Our sample also included taxa from non-erect outgroups (*Ornithorhynchus* and *Tachyglossus* for eutherian mammals, *Alligator mississippiensis* for saurischian dinosaurs). We chose these taxa specifically to test if posture influenced subchondral shape scaling in much the same way it does for overall long bone dimensions. In other words, by including taxa with homologous epiphyseal joints but non-parasagittal postures, we could test how much subchondral shape scaling was influenced by long bone orientation. It should be added that as our goal was to track subchondral bone shape scaling associated with gigantism in eutherian mammals and non-avian saurischian dinosaurs, our outgroup taxa were not selected to be an exhaustive phylogenetic sample of all possible variants. In both samples, the outgroup taxa served primarily as smaller-bodied specimens that helped us constrain and root our analysis of subchondral scaling trends in mammalian and dinosaurian giants in a broader context.

### Permission and Institutional Abbreviations

Permission was obtained to examine specimens at the following institutions: AMNH, American Museum of Natural History, USA; BP, Bernard Price Institute for Palaeontological Research, University of Witwatersrand, Johannesburg, South Africa; BYU, Brigham Young University, USA; CM, Carnegie Museum of Natural History, USA; DNM, Dinosaur National Monument, USA; FMNH, Field Museum of Natural History, USA; MWC, Museum of Western Colorado, USA; OMNH, Oklahoma Museum of Natural History, USA; SAM, Iziko South African Museum of Natural History, Cape Town, South Africa; SMNS, Stuttgart Museum of Natural History, Germany; UMNH, Utah Museum of Natural History, USA; USNM, United States Museum of Natural History (Smithsonian), USA; WIU, Western Illinois University, USA; YPM, Yale Peabody Museum, USA. See Tables S1 and S2 for specimens examined. No specimens were loaned, destructively sampled, or purchased.

### Metrics

Length measurements were made with digital calipers, tree calipers, or metric tape to the nearest millimeter. The measurements were subsequently log10 transformed to normalize their distribution [35]. For GM analysis, we used two-dimensional thin-plate splines (TPS) because this technique is ideal for analyzing a set of objects (long bones) that are similar in overall morphology and where the detection of more subtle shape differences is desired [36], [37]. In a TPS analysis, homologous landmark coordinates of all specimens are aligned, rotated, and scaled into a grand mean reference form via Generalized Least Squares (GLS) Procrustes superimposition [36], [37]. Measuring the sum of squared Procrustes distances in the homologous landmark coordinates of each specimen against the reference form reveals shape differences which can be analyzed mathematically and visualized as a deformation grid or thin-plate spline [38]. Normalized shape coefficients generated from the sum of squared Procrustes distances (partial warps) are correlated, dependent variables that collectively describe shape and are analyzed with standard multivariate statistics [36], [37]. A principal components analysis (PCA) of the partial warps using a variance-covariance matrix is then used to produce relative warps or principal components (PRINs) of shape that can be tested statistically and visualized using deformation grids. Interested readers are referred to the extensive literature on the mechanics and mathematical theory underlying thin-plate splines [36]–[38] as well as applied biological and paleontological examples of TPS [14], [26], [39], [40].

**Figure 1:**
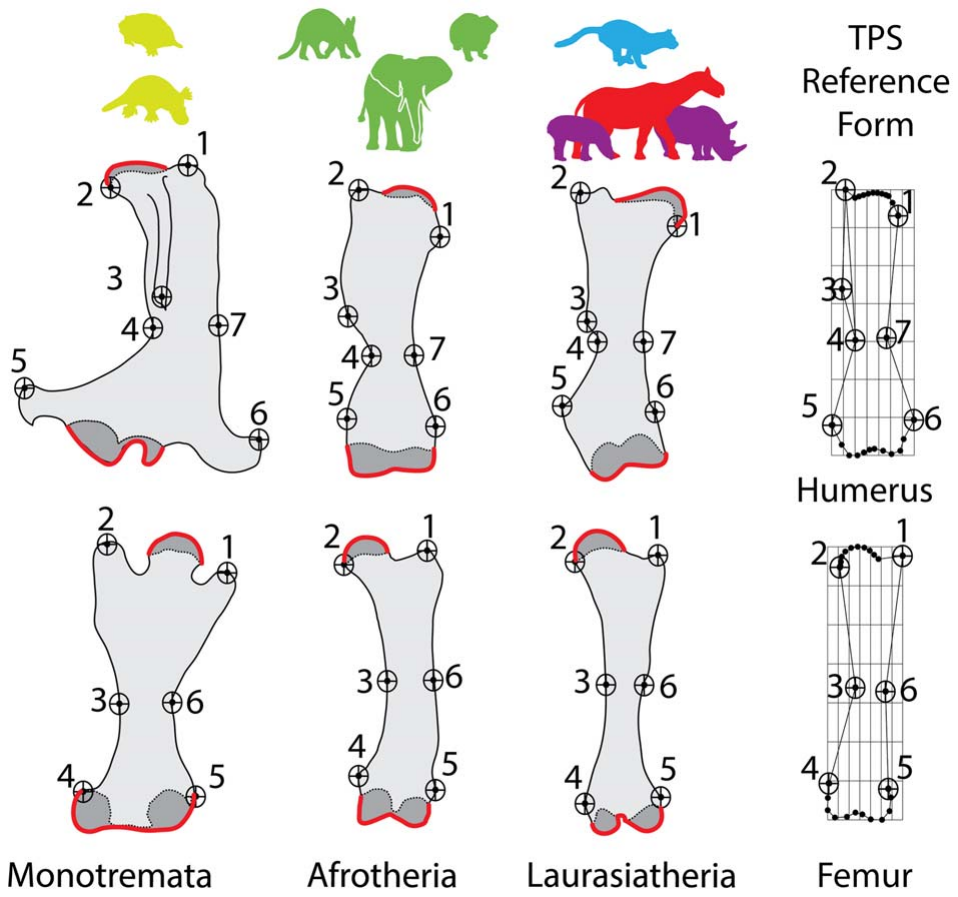
Landmarks digitized for 2-D thin-plate splines (TPS) geometric morphometrics (GM) analyses – Mammals. All drawings are not to scale and were enlarged or reduced to facilitate comparisons. For the humerus, landmarks 1 and 2 encompass the region between the greater tubercle and the medial extent of the humeral head. Landmarks 2, 3, and 4 encompass the extent of the deltopectoral crest. Landmarks 4 and 7 encompass the narrowest point of the midshaft. Landmarks 5 and 6 denote the lateral and medial epicondyles, respectively. For the femur, landmarks 1 and 2 encompass the region between the greater trochanter and the medial extent of the proximal end of the femur. Landmarks 3 and 6 encompass the narrowest point of the midshaft. Landmarks 4 and 5 denote the the lateral and medial epicondyles. Regions of sub-articular bone are indicated in dark gray and the digitized outlines that were subsequently converted into chains of 10 evenly-spaced semi-landmarks are colored red. The exemplar taxa represented as bones, left to right, are: *Ornithorhynchus*, *Mammut*, and *Paraceratherium*. The TPS reference forms which describe the shape of the bones mathematically is shown at right.

**Figure 2:**
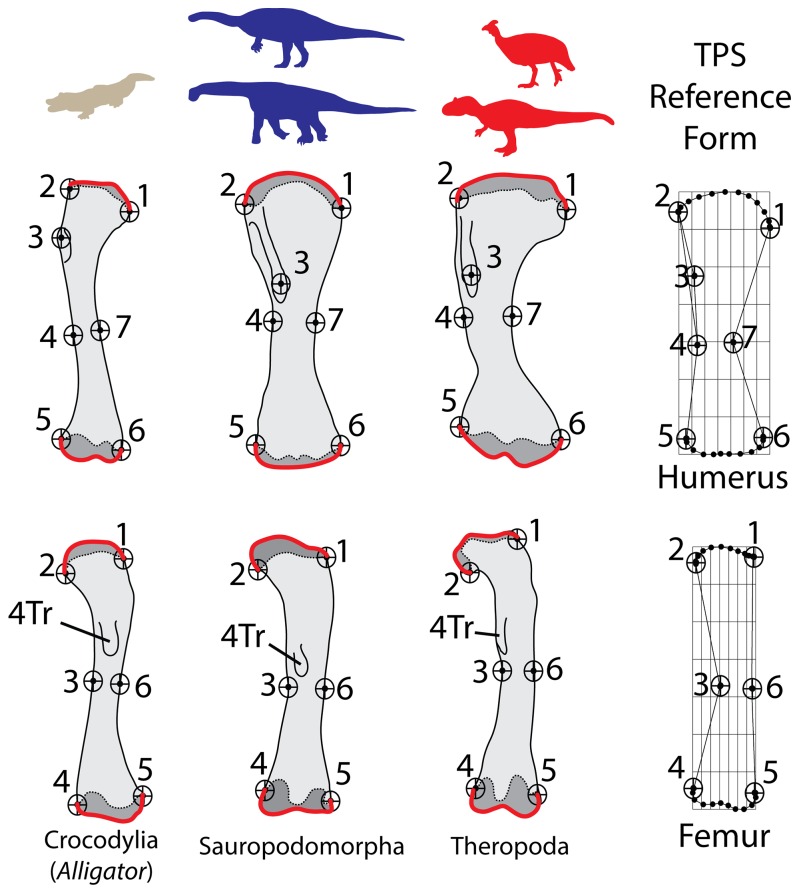
Landmarks digitized for 2-D thin-plate splines (TPS) geometric morphmetric (GM) analyses – Archosaurs. All drawings are not to scale and were enlarged or reduced to facilitate comparisons. For the humerus, landmarks 1 and 2 encompass the region of the humeral head. Landmarks 2, 3, and 4 encompass the extent of the deltopectoral crest. Landmarks 4 and 7 encompass the narrowest point of the midshaft. Landmarks 5 and 6 denote the lateral and medial epicondyles, respectively. For the femur, landmarks 1 and 2 encompass the region of the femoral head. Landmarks 3 and 6 encompass the narrowest point of the midshaft. Landmarks 4 and 5 denote the the lateral and medial epicondyles. Regions of sub-articular bone are indicated in dark gray and the digitized outlines that were subsequently converted into chains of 10 evenly-spaced semi-landmarks are colored red. The exemplar taxa represented as bones, left to right, are: *Alligator*, *Torneria* (“*Barosaurus*”), and *Allosaurus*. Given that birds were included in the Archosauria sample, the fourth trochanter (4 Tr) was not digitized. The TPS reference forms which describe the shape of the bones mathematically is shown at right.

Mammal and archosaur long bones were digitized and analyzed using the TPS program suite [41]. Procedures for capturing the 2-D images of the long bones and the landmarks selected for digitization followed standards detailed elsewhere [14], [15], [31]. Figures 1 and 2 provide illustrations of the selected landmarks for digitization. Given that our sample included bird femora lacking a fourth trochanter, this particular landmark was not digitized in our archosaur sample so that all specimens were comparable (Figure 2). *Deinotherium* and the titanosaur sauropod taxa (Tables S1 and S2) were digitized from published photos. Utilizing photographic images from other sources can potentially introduce sources of error related to different camera angles and focal lengths controlled for in our samples. However, all photos selected were taken of the appropriate sides and orientations of these bones, and the morphology we were interested in capturing largely falls within a two-dimensional plane (see below). Moreover, we ran the analyses presented here with and without these photographs, and no significant differences in the signal or outcomes of our results were affected. Therefore, we chose to include these photographic specimens that, while not ideal, significantly added to the phylogenetic range of our dataset.

Changes in limb bone morphology associated with landmarks are indicated by numbers in parentheses in the text. Sliding semi-landmarks were generated from curves that captured the two-dimensional outline of the subchondral surfaces in a mediolateral plane [36]. For each specimen, outlines of the subchondral surfaces were converted into a chain of 10 equally-spaced semi-landmarks using the program TPSUtil [41]. See Figure 1 for an illustrated description of the major landmarks selected for TPS analysis.

A PCA of the partial warps was run to determine where the most shape variation occurred in the sample, expressed as PRIN 1. This was done to determine how long bone and subarticular shapes changed among taxa and postures irrespective of size. The program TPSRelw generated the deformation grids for visualization of these shape changes. To determine whether there were significant differences among the taxa or postures in relation to shape, a MANOVA of the partial warps was first performed. This test only confirms whether or not there are significant differences in the sample, but cannot be used for pairwise comparisons used to distinguish among which taxa or postures these differences are occurring. This is because the partial warps are dependent variables that collectively describe shape, and so variation in individual partial warps is biologically meaningless. Instead, following the approach taken in several recent studies (e.g., [42]), we analyzed PRIN 1 for significant differences using the non-parametric Kruskal-Wallis test followed by pairwise Mann-Whitney U tests to tease apart where and how differences in long bone and subarticular shapes were occurring.

Next, regression of partial warps onto log-transformed humerus and femur length was used to determine how subchondral bone shape scaled with size. Multiple regression analyses of the partial warps on log10-transformed humerus and femur length were used to determine how well the data fit a linear model, generating multiple r^2^ values which show how well or how poorly the predicted subchondral shape change of the humerus or femur fits the data. Centroid size, rather than a linear size measurement (i.e., humerus or femur length), is often used in geometric morphometrics because it represents a pure geometric scaling variable with mathematical independence from shape [36], [38]. However, length was chosen instead of centroid size to simplify comparisons of these results with previous, non-geometric morphometric studies. Moreover, humerus and femur length are variables well-correlated with the mechanical properties of long bones [18], [43] as well as with body size [31], [32].

Multivariate regressions of the partial warps on log10-transformed humerus and femur length were used to test for allometry and to produce thin-plate splines deformations. Unlike linear regression, geometric morphometric (shape) allometry is not reported as a slope value. Instead, against a null model of isometry (i.e., no significant shape difference occurs between the specimens and the mean with increasing length), a significant, multivariate difference (*p*<.05 based on Goodall’s F-test) in partial warp scores from the predicted mean indicates humerus or femur subchondral allometry (significant shape change). Goodall’s F-test, a statistic designed specifically for geometric morphometric regression [44] was used to generate a coefficient of determination (R^2^) which, following Monteiro [40], is expressed as a percentage of shape change explained by increasing humerus and femur length. The higher the percentage expressed in the R^2^ value, the greater influence size has on shape. A multivariate regression analysis using Wilk’s λ was used to determine if the coefficient of determination and deformation grids reflected a significant allometric trend. This statistical analysis was run in the TPSRegr program.

### Caveats

The bony landmarks and subchondral surfaces of mammals and dinosaurs are not directly homologous in some cases. For example, the region identified as the lesser trochanter on the femur in mammals is not directly homologous to this same region in archosaurs [45]. Moreover, the subchondral surfaces of mammals and archosaurs differ in that, for mammals, these surfaces are only ever covered by articular cartilages, whereas in archosaurs some regions of these areas are also associated with muscular insertions (see for example Petermann and Sander [46]). Therefore, whereas we are comparing the scaling trends between mammals and archosaurs, we did not pool our mammal and archosaur data. Instead, separate analyses of these groups were performed to ensure that the trends we report were not biased by conflation of homologous and non-homologous landmarks or by regions of semi-landmarks that, in archosaurs, are also associated with additional soft tissues.

Given that our data for the subchondral shape in archosaurs encompasses both articular cartilages and fibrocartilages associated with other soft tissues, how can we be sure that we are not just reporting trends in the expansion of the metaphysis or that our data are comparable to those of mammals? The objective of our study was neither to reconstruct accurate joint surfaces, nor simply to measure proximal and distal long bone widths, but rather to explore how the subchondral bone shape itself scales with size. We are testing the hypothesis that different shape scaling trends should be readily apparent given the acknowledged differences in chondroepiphyseal thickness and composition between mammals and archosaurs. Thus, differences we detect in subchondral shape (not simply width) remain informative and indicative of differences in its adaptive role in parlaying dynamic locomotor stresses absorbed through the overlying chondroepiphysis.

Why did we use a two-dimensional analysis instead of a three-dimensional analysis? Undoubtedly, the capture and analysis of three-dimensional shape would further enhance our analysis and assist us in interpreting patterns of subchondral bone shape. However, a number of challenges prevented such an approach. First and most significantly, the data collected in this study span a period of over 10 years during which time cost-effective and portable three-dimensional scanning technologies for acquiring large bone geometries have only recently started to become available. Had we access to these technologies ten years prior, we would have utilized them, as we plan to utilize such approaches in future studies. Second, our main goal in this study was to quantify whether or not there were significant differences in the scaling patterns of surface morphology between eutherian mammal and saurischian dinosaur long bones, and whether such differences were correlated with known differences in articular cartilage properties. Again, our goal was not to realistically recreate joint surfaces or establish precise measures of joint articulation, nor do we propose how the three-dimensional shape of the subchondral bone is used to reconstruct joint geometry. Our selection of the humerus and femur furthers our goal: these are long bones in which a significant portion of the subarticular surfaces can be reliably captured and interpreted in two dimensions [14], [26]. In fact, several previous two-dimensional analyses of both non-avian dinosaur and extant archosaur long bones using the same orientations and techniques have revealed significant, biologically-relevant patterns that have informed the on-going discussion and interpretation of dinosaur locomotion [14], [15], [26], [31]. Finally, third, two-dimensional data is valuable, comparable to previous studies, and provides a good first-level approximation of scaling patterns. Just as linear morphometrics informed and directed the study of two-dimensional GM of long bones, so, too, can two-dimensional GM illuminate where future three-dimensional GM studies can make the best impact. Our study is certainly not the last word on long-bone scaling and subarticular patterns in non-avian dinosaurs. Rather, we hope it inspires and provides the basis for research incorporating three-dimensional technologies in years to come.

## Results

**Figure 3:**
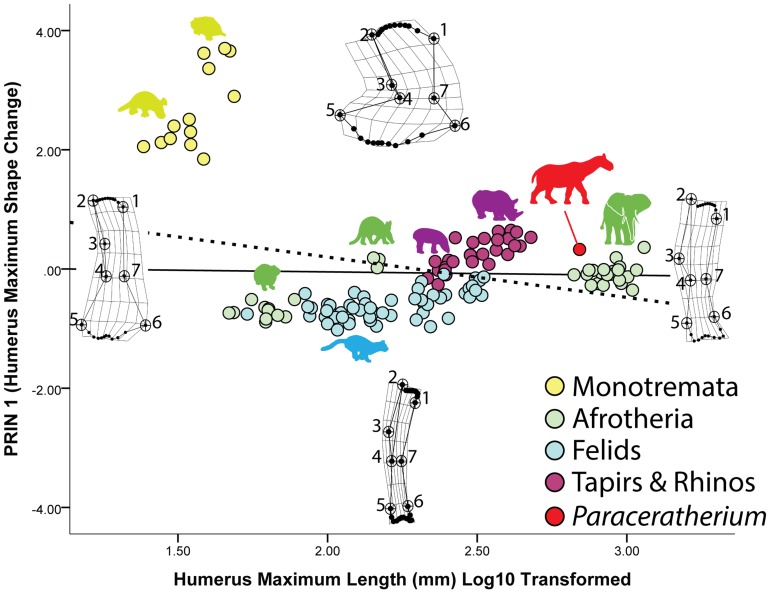
Changes in humerus shape in mammals. Maximum humerus shape change in the sample is shown on the Y-axis (PRIN 1), whereas humerus shape changes associated with size are shown on the X-axis. Note that on the PRIN 1 axis, monotreme taxa plot separately from other mammals in the sample, and show a much more expanded and robust humerus. On the X-axis, the sub-articular bone region narrows significantly with increasing size, and the shapes of these regions become more convex and/or distinct.

A PCA of the partial warps of the mammalian humerus reveals, as might be predicted, distinct differences in shape between monotremes and eutherians (Figure 3). The PRIN 1 axis describes various aspects of humerus shape including the width of the proximal end (landmarks 1,2), the orientation of the deltopectoral crest (landmarks 2, 3, 4), and the breadth of the region lying between the lateral and medial epicondyles (5, 6) (Figure 3). Humeri that plot more positively along the PRIN 1 axis become wider proximally and distally and possess a medially-inflected deltopectoral crest, whereas those which plot negatively show greatly narrowed proximal and distal ends with a laterally-oriented deltopectoral crest (Figure 3). A MANOVA of the partial warps shows that there is a significant difference among the taxa (Table 1), and subsequent KW and MWU tests on PRIN 1 indicate that each taxonomic grouping has a humerus shape significantly different from one another (Table 2). The monotreme taxa all plot positively along the PRIN 1 axis, and this reflects their rather wide and robust humeri. All the eutherian mammals in the sample fall close to the reference form and may plot somewhat negatively or positively on the PRIN 1 axis. Reflecting previous studies (e.g., [47]), ceratotherians (except for *Paraceratherium*) have the most robust humeri among the eutherian taxa, whereas proboscideans and *Paraceratherium* have narrower, more gracile humeri. Perhaps not surprisingly, all felids plot negatively along PRIN 1, reflecting their relatively gracile humeral morphology. Curiously, among all the eutherian mammals in the sample, whereas significant differences in shape exist, variation in humerus morphology is relatively limited. This is reflected in the KW test for posture that showed a significant difference between monotremes (non-erect) and the eutherians (erect) in the sample (Table 3). However, this shape difference is not associated with the subchondral bone (see below) and instead appears to reflect the expansion of the epicondyles (landmarks 5, 6) and relatively “squat” humeri of the monotremes (Figure 3).

**Table 2:** Kruskal-Wallace and pair-wise Mann-Whitney U non-parametric tests for significant differences in maximum shape change (PRIN 1) among the sampled taxa based on the median score of PRIN 1.

Taxon	Element	n	Median	KW Statistic	df	Asymptotic p	Mann-Whitney U
Saurischia*+*Alligator*	Humerus 62.5%	100	−0.128	25.237	2	<0.0001	Sauropodomorpha
Saurischia*+*Alligator*	Femur 50%	97	−0.131	68.960	2	<0.0001	All
Eutheria**+Monotremata	Humerus 63%	132	−0.244	74.961	3	<0.0001	All
Eutheria**+Monotremata	Femur 65%	127	−0.341	83.330	3	<0.0001	All

Percentages listed with each element show how much shape variation in the sample PRIN 1 accounts for. Significant differences among the taxa sampled (p<0.05) are indicated under the Mann-Whitney U column.

* Saurischia was divided into Sauropodomorpha and Theropoda (Aves inclusive).

** Eutheria was divided into several clades: Afrotheria, Perissodactyla (rhinos and tapirs only), and Felidae.

**Table 3:** Kruskal-Wallace and pair-wise Mann-Whitney U non-parametric tests for significant differences in maximum shape change (PRIN 1) among the postures of the sampled taxa based on the median score of PRIN 1.

Taxon	Element	n	Median	KW Statistic	df	Asymptotic p	Mann-Whitney U
Saurischia*+*Alligator*	Humerus	100	−0.128	16.183	2	<0.0001	*Alligator*
Saurischia*+*Alligator*	Femur	97	−0.131	48.291	2	<0.0001	Sauropoda
Eutheria**+Monotremata	Humerus	132	−0.244	14.420	1	<0.0001	Monotremata
Eutheria**+Monotremata	Femur	127	−0.341	14.256	1	<0.0001	Monotremata

Significant differences among the taxa sampled (p<0.05) are indicated under the Mann-Whitney U column. Posture categories were: 1) non-parasagittal; 2) bipedal, parasagittal; and 3) obligate quadrupedal, parasagittal.

* Saurischia was divided into Sauropoda (parasagittal obligate quadrupeds) and parasagittal bipeds (Theropoda (Aves inclusive)+Basal Sauropodomorpha).

** Eutheria was treated as one postural category: parasagittal obligate quadrupeds.

When the partial warps of the mammalian humerus are regressed on their log10 transformed length, size and shape are shown to be well-correlated and statistically significant (Table 4). Size-related shape changes in the humerus account for 13% of the variation in the sample. Deformation grids show that as humerus size increases, the proximal (landmarks 2, 3) and distal ends (landmarks 5, 6) of the humerus become narrowed, and that the deltopectoral crest (landmarks 2, 3, 4) becomes more laterally-oriented (Figure 3). However, surprisingly the subchondral surfaces remain narrow or become narrower with increasing size; these regions even remain relatively narrow in the monotreme sample. Proximally, the humeral head becomes slightly more convex and medially-oriented (semi-landmarks between landmarks 2 and 3), whereas distally the regions of subchondral bone underlying the capitulum and trochlea (lateral and medial condyles) become more distinct and convex (semi-landmarks between landmarks 5 and 6) (Figure 3).

**Table 4:** Multiple and multivariate regression of partial warps on size (humerus or femur maximum length).

Taxon	Element	n	Mult-r^2^	Wilk’s λ	F	df	p	R^2^
Saurischia+*Alligator*	Humerus	100	0.8871	0.11285347	7.704	50.00, 49.00	<0.0000001	**29.7%**
Saurischia+*Alligator*	Femur	97	0.9034	0.09656473	9.356	48.00, 48.00	<0.0000001	**23.6%**
Eutheria*+Monotremata	Humerus	132	0.9020	0.09799221	14.912	50.00, 81.00	<0.0000001	**12.7%**
Eutheria*+Monotremata	Femur	127	0.8809	0.11910716	12.018	48.00, 78.00	<0.0000001	**5.1%**

Multiple r^2^ (Mult-r^2^) values indicate how well the shape changes correlate with size. R^2^ a coefficient of determination that is expressed as a percentage of shape change explained by length.

**Figure 4:**
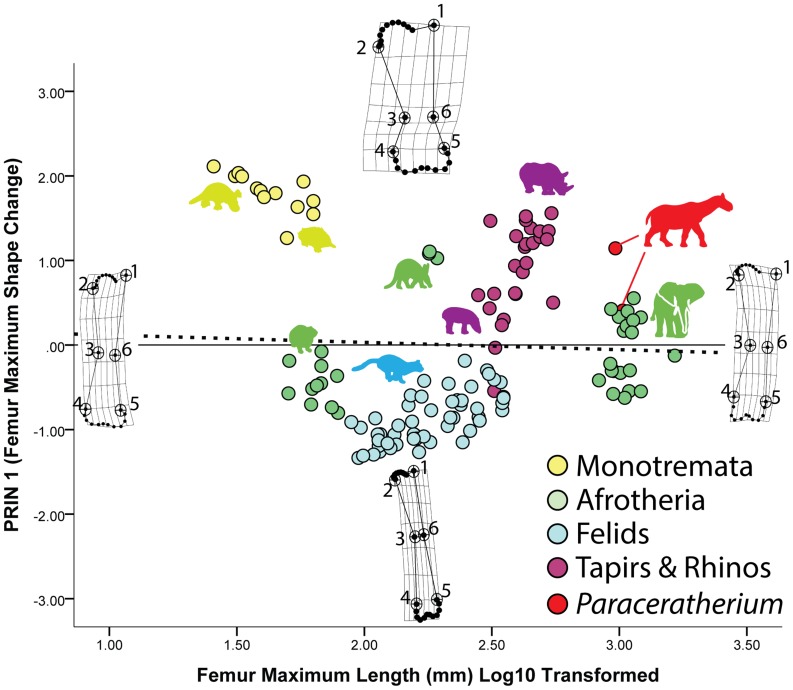
Changes in femur shape in mammals. Maximum femur shape change in the sample is shown on the Y-axis (PRIN 1), whereas femur shape changes associated with size are shown on the X-axis. As with the humerus, monotreme specimens plot among the most robust femora on the PRIN 1 axis, but aardvarks, rhinos, and one *Paraceratherium* specimen also plot in robust morphospace. Once again, the sub-articular bone region narrows significantly with increasing size and that the shapes of these regions become more defined, convex, and/or distinct. Notice also that the femoral head becomes more distinct and medially-oriented with increasing size.

The PCA of the mammalian femur reveals starker trends and differences among the taxa along the PRIN 1 axis. For the femur as with the humerus, the PRIN 1 axis describes changes in girth and robustness. Specimens plotting positively along this axis show trends towards expanding breadth at their proximal (landmarks 1, 2) and distal ends (landmarks 4, 5) as well as at their midshaft (landmarks 3, 6). Conversely, specimens plotting negatively along the PRIN 1 axis show distinct narrowing proximally (landmarks 1, 2), distally (landmarks 4, 5), and at midshaft (landmarks 3, 6) (Figure 4). A MANOVA of the partial warps revealed a significant difference in shape among the taxa (Table 1), and subsequent KW and MWU tests show that all mammalian taxa have significantly different femoral shapes (Table 2). A strong allometric trend toward robust femora is starkly revealed among the ceratotherians. Here again, such robust allometry has previously been reported for the linear dimensions of these taxa [47]. Curiously, of the two *Paraceratherium* specimens, one plots highly positively along PRIN 1 with the other ceratotherians, but the other plots among the proboscideans which straddle the reference form and plot both positively and negatively on this axis (Figure 4). The femora of *Orycteropus* are fairly robust, whereas those of hyraxes are more gracile. As with the humerus, felids show more gracile femora, plotting without exception along the negative axis of PRIN 1 (Figure 4). Moreover, a KW test on PRIN 1 comparing posture (non-erect versus parasagittal) shows a significant difference in femoral shape between monotremes and the eutherian mammals in the sample (Table 3). As with the humerus, monotremes have robust femora with slightly expanded epicondylar regions (landmarks 4, 5) (Figure 4), but again these trends do not seem to correlate with the relatively narrow subcondral bone regions (landmarks 1, 2 and the intervening semi-landmarks).

Regression of the partial warps onto log10 transformed femur length indicates that size and shape are well-correlated (Table 4), but that only 5.1% of the shape change in the sample is size-related. Most of the size-related changes in femur shape are correlated with subchondral bone shape. As the mammal femora in the sample become larger, the region of the femoral head (the semi-landmarks between landmarks 1 and 2) becomes narrower and more convex, whereas the subchondral surface underlying the distal condyles (the semi-landmarks between landmarks 4 and 5) becomes divided into two distinct, convex regions (Figure 4).

**Figure 5:**
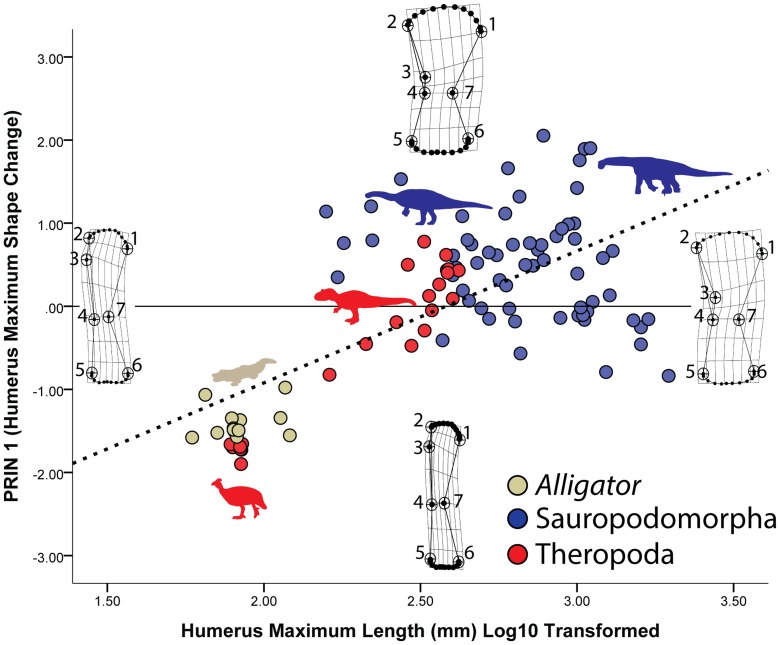
Changes humerus shape in saurischian dinosaurs and alligators. Maximum humerus shape change in the sample is shown on the Y-axis (PRIN 1), whereas humerus shape changes associated with size are shown on the X-axis. Changes in humerus shape along the PRIN 1 axis and X-axis are similar in that larger taxa have more proximally and distally expanded ends. In particular, the sub-articular region expands tremendously whereas its overall shape remains gently convex. Note also that the deltopectoral crest (landmarks 2–4) remains or becomes more medially-deflected as size increases.

A PCA of the partial warps of the Saurischian dinosaur and *Alligator* humerus sample (archosaur sample hereafter) shows a pattern different from that reported for the mammals. As with the mammal sample, the PRIN 1 axis describes expansion and robustness: specimens that plot positively along this axis show expanded proximal (landmarks 1, 2) and distal (landmarks 5, 6) ends as well as a lengthening and more medially oriented deltopectoral crest (landmarks 2, 3, 4) (Figure 5). A MANOVA of the partial warps shows that humerus shape differed significantly among the taxa in the sample (Table 1), and subsequent KW and MWU tests on PRIN 1 confirm that sauropodomorph taxa have humeri significantly different in shape from those of theropods and *Alligator* (Table 2). Confirming previous findings [14], [15], the humeri of sauropod taxa show a large degree of variation in humerus robustness, with the Macronarians in the sample trending toward more gracile humeri with increasing size (Figure 5). However, when categorized by posture, *Alligator* humeri were significantly different from those of the non-avian dinosaurs, although it is not clear why (Figure 5; Table 3).

Regression of the partial warps on log10 transformed humerus length shows that size and shape are indeed well-correlated, with size-related shape changes accounting for nearly 30% of the shape variation (Table 4)! Surprisingly, the size-related shape trend is nearly identical to that recorded along the PRIN 1 axis: with increasing size, the humerus expands mediolaterally (landmarks 1, 2; 4, 7; 5, 6) and the deltopectoral crest lengthens and shifts medially (landmarks 2, 3, 4) (Figure 4). Thus, unlike the mammal sample, shape changes along PRIN 1 and those due to size are interrelated, and PRIN 1 is perhaps best described as a size and shape component. The subchondral bone proximally (the semi-landmarks between landmarks 1 and 2) and distally (the semi-landmarks between landmarks 5 and 6) changes little in shape with increasing size, but it does become slightly more convex in larger specimens (Figure 5). As with the mammal sample, subchondral shape trends remain consistent with increasing size despite other changes related to posture and phylogeny.

**Figure 6:**
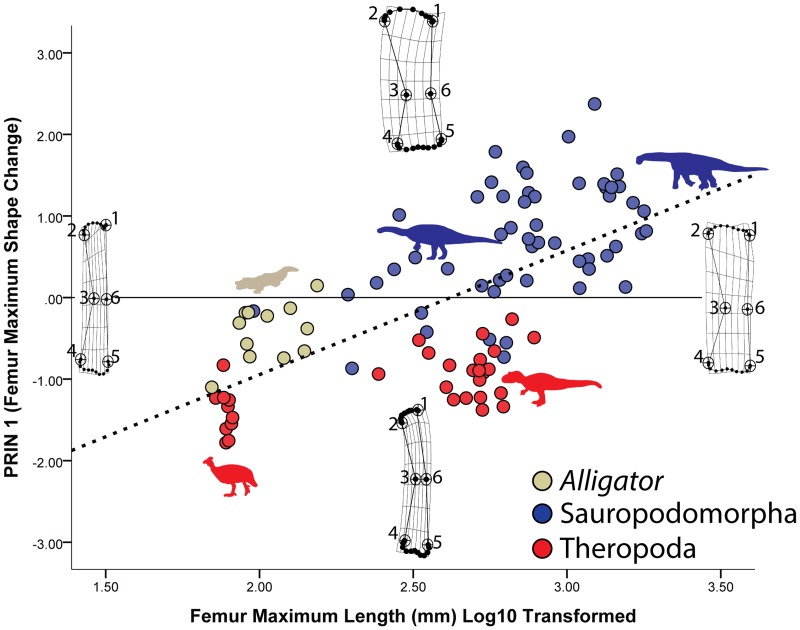
Changes in femur shape saurischian dinosaurs and alligators. Maximum femur shape change in the sample is shown on the Y-axis (PRIN 1), whereas femur shape changes associated with size are shown on the X-axis. As with the humerus, changes in femur shape along the PRIN 1 axis and X-axis are similar in that larger taxa have more proximally and distally expanded ends. Overall, the sub-articular region expands tremendously whereas its overall shape remains gently convex, although the distal condyles become somewhat more pronounced.

The pattern of shape changes for the archosaur femur sample is remarkably consistent with the humerus. A PCA of the femur partial warps yields a PRIN 1 that again shows increasing robustness as well as proximal (landmarks 1 and 2) and distal (landmarks 4 and 5) expansion (Figure 6). Subsequent KW and MWU tests show that all archosaur taxa in the sample have significantly different femur shapes (Tables 1 and 2). Unlike the humerus sample, theropod femora appear to remain more gracile for a given size, whereas those of alligators and sauropodomorphs tend to be more robust (Figure 6). When KW and MWU tests of PRIN 1 were run for posture, sauropods show a significantly different femur shape when compared to *Alligator*, theropods, and basal sauropodomorphs (Table 3). This shape difference appears to correlate with the overall expansion of all regions of the sauropod femur (Figure 6).

The regression of the partial warps onto log10 transformed femur length again shows that size and shape are well-correlated, and that over 23% of the shape variation in the sample is correlated with size (Table 4). As with the humerus, much of the size-related shape variation again reflects the trends reported for PRIN 1: increasing robustness and expansion of the proximal (landmarks 1, 2) and distal (landmarks 4, 5) ends (Figure 6). Again, the subchondral surfaces of the archosaur femur expand and become slightly more convex. At the femoral head, the subchondral surface gently becomes more convex medially (semi-landmarks between landmarks 1 and 2), whereas the distal subchondral surface forms two gently convex regions corresponding to the distal condyles separated by a shallow divot (semi-landmarks between landmarks 4 and 5) (Figure 6). Again, subchondral shape trends remain consistent across the size range in the sample.

## Discussion

Our results show that despite differences in posture and phylogeny, there is a clear and significant signal in the data related to subchondral shape scaling. For our mammal sample, whereas many variations in shape occur among the different clades sampled and between non-erect and parasagittal postures, only minor changes to subchondral shape occur with increasing size (Figures 3 and 4). Typically, with increasing size the subchondral regions become relatively narrower, the humeral and femoral head regions become more convex, and the distal subchondral regions form distinct, convex condyles. In contrast, the archosaur sample shows a trend towards overall expansion of the subchondral surfaces proximally and distally with increasing size (Figures 5 and 6). Whereas larger humeri and femora show trends toward increasing convexity, these trends are more subtle than for the mammal sample and the appearance of distinct, distal condyle regions only appear in subdued form on the distal end of the femur. These results support our hypothesis that there are significant, shape scaling differences in the subchondral regions of the humerus and femur between eutherian mammals and saurischian dinosaurs.

Could the use of bipedal taxa in the saurischian sample (theropods, basal sauropodomorphs) affect the scaling patterns we report, especially for the humerus? Whereas as this is certainly possible, our results for these archosaurs show no particular deviation among bipeds or quadrupeds with increasing size (see Figures 4 and 5, and Table 3). Moreover, for the humerus where such a difference in posture between bipedal saurischians and sauropods would be predicted to occur, a significant difference is instead reported for alligators (see Table 3). Therefore, we have no evidence that differences in posture related to bipedalism had a significant effect on our data and its interpretation.

Our results suggest that subchondral shape scaling patterns reflect relative articular cartilage thickness. For mammals articular cartilage remains relatively thin across all size ranges (a few millimeters in thickness), even for elephants [48], [49]. In fact, the relative thickness of articular cartilage decreases with increasing size in eutherian mammals [48]. In contrast, archosaurs, presumably including non-avian saurischian dinosaurs, retain or retained much thicker articular cartilage (in some cases >1 cm) throughout their lives and at large size [25]. Although at least some archosaurs show a relative decrease in articular cartilage thickness with increasing body mass [26], this tissue nevertheless remains much thicker than reported for mammals of similar mass. In fact, two recent, independent studies have verified that as much as 10% or more of long bone length in extant archosaurs (and presumably non-avian saurischian dinosaurs) can be comprised of articular cartilage [25], [26]!

How do these data support our suggestion that subchondral shape scaling patterns reflect articular cartilage thickness? Bonnan and colleagues [26] showed that as alligators and two bird species grew larger, not only did the relative amount of their articular cartilage decrease significantly, but that the underlying subchondral bone began to resemble the shape of the overlying articular cartilage. In fact, for the femur in alligators and birds, and for the humerus in guineafowl (*Numdia*), no significant difference between subchondral bone shape and articular cartilage shape were reported in larger individuals and adults [26]. In other words, as articular cartilage became relatively thinner with increasing size, the shape of the subchondral bone and articular cartilage became statistically indistinguishable. Perhaps not surprisingly, subchondral bone shape in eutherian mammals very clearly resembles the shape of the relatively thin overlying articular cartilage. This is certainly not surprising to human and mammal anatomists who can typically assume that the way dry bones articulate is a close approximation for their *in vivo* orientations. Thus, thinner articular cartilage would appear to be associated with a subchondral bone shape displaying well-developed surfaces, condyles, and convexity. In contrast, as shown by Bonnan et al. [26] for extant archosaurs, thicker articular cartilage is associated with flatter, poorly-developed surfaces and a less convex shape. Here, too, this observation is not surprising to those who work with archosaur long bones and the many uncertainties that arise from the relative lack of articular data [25], [26], [50]. Thus, we conclude that subchondral bone which is relatively narrow in relation to the metaphysis and which displays pronounced geometry and convexity is strongly correlated with thin articular cartilage. In contrast, subchondral bone that remains relatively broad in relation to the metaphysis and which is relatively flattened and bereft of distinct geometry or convexity is strongly correlated with thick articular cartilage.

The inescapable conclusion from our data is that saurischian dinosaurs frequently achieved gigantism while retaining joints comprised of relatively thick articular cartilage, a claim that bolsters recent work and similar suggestions by Holliday and colleagues [25]. This is an astounding conclusion given that the largest known sauropod femora measure well over 2 meters in length [51], [52] and mass estimates for many sauropods exceed 20,000 kg [6], [7], [9]! In contrast, although certain eutherian mammal taxa have achieved gigantism (proboscideans and *Paraceratherium*, maximum known femur lengths of approximately 1.7 m and 1.2 m, respectively [11], [53]), it was much rarer [7], [8]. Our data also suggest that this difference in articular cartilage thickness is not a function of posture: our reported size trends showed no significant correlation with an erect or non-erect stance. Instead, where significant differences in posture are detected, they are correlated with other regions of the bone associated with muscle insertion (epicondyles) or weight support (shaft thickness) (Figures 3–6). Thus, our data strongly suggest that terrestrial gigantism in both mammals and saurischian dinosaurs is correlated, at least to some degree, with articular cartilage thickness, regardless of posture.

It is well-established that bones change their shapes to best resist dynamic loads [23], and subchondral bone should be no different. Recent data on trabecular bone scaling support this inference. In mammals, birds, and crocodylians, the trabeculae of subchondral bone scale allometrically with increasing size [54]. In particular, in larger mammals and birds bone remodeling results in fewer but thicker trabeculae, a strategy that appears to diminish strain [54]. Therefore, inferring that subchondral shape is correlated with subchondral adaptations to dynamic loads follows previously established trends for this region. Given the relative differences in articular cartilage thickness between eutherian mammals and saurischian dinosaurs, it is not surprising that the shape scaling trends in the subchondral bone should also differ.

**Figure 7:**
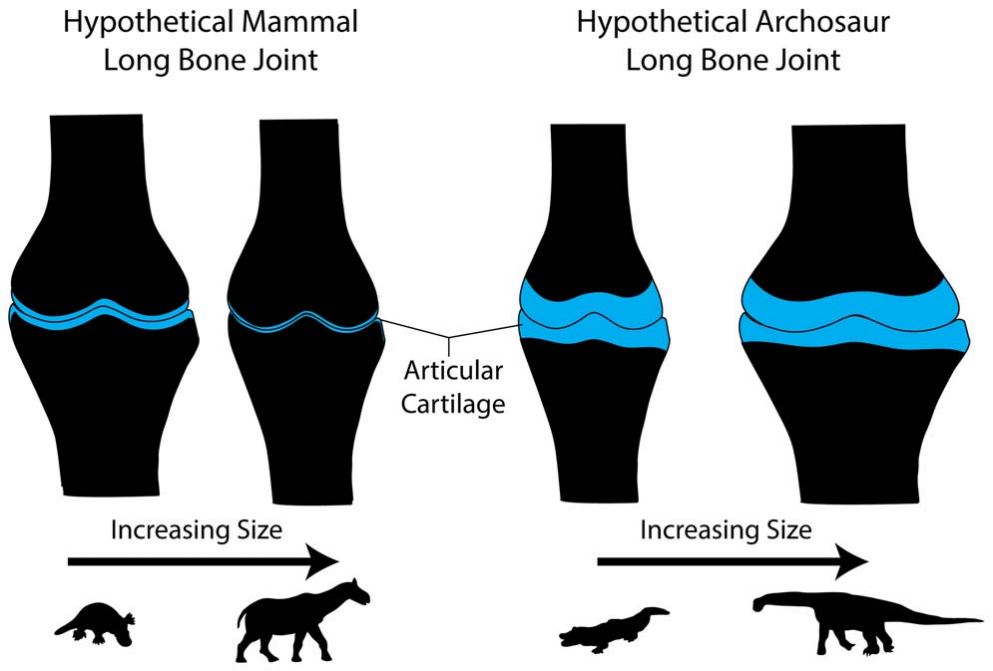
Schematic representation of changing joint surfaces and articular cartilage thickness with increasing size in mammals and archosaurs. Our data suggest that as mammals increase in size, the joint region narrows and the articular cartilage becomes relatively thinner, producing convex and well-developed subchondral bone surfaces. For archosaurs, our data suggest that with increasing size the joint region expands while the articular cartilage remains thick, producing relatively flat and less convex subchondral bone surfaces.

It has been demonstrated that when articular cartilage is thin, stress is best dissipated by forming more and more congruent joints [55], [56]. That is, the load or force being imposed on a joint is best dispersed by having closely articulating surfaces that spread stress over much of the epiphysis [55], [56] and into the underlying subchondral bone. Moreover, congruent joints ensure that the articular cartilage is loaded in predictable patterns which may limit shear stresses or inhibit peak, focused pressure points [49], [57]. In fact, enhanced joint congruence in eutherian mammals such as dogs [57] and elephants [49] is well-documented, and typically joint congruence increases with increasing size [58]. Furthermore, the amount of joint congruence varies inversely with cartilage thickness in eutherian mammals. For example, the elbow in mammals is one of the most congruent joints, and this is where articular cartilage is typically thinnest, whereas somewhat less joint congruence is present at the knee where cartilage is often thickest [55]–[57], [59]. Thus, the formation of distinct and often convex subchondral surfaces would be predicted because these shapes would best support thin articular cartilage and enhance joint congruence. We find it significant, therefore, that the subchondral surfaces of the eutherian mammal long bones in our sample scale such that they become narrow and well-defined with increasing size, trends that are consistent with the maintenance of thin articular cartilage and extensively congruent joints (Figure 7).

The picture that emerges for archosaurs and saurischian dinosaurs in particular is a bit more complex. Certainly, although articular cartilage was significantly thicker than in mammals, there is no doubt that the articular surfaces themselves formed congruent joints. In fact, that the thick, cartilaginous articular cartilages articulated in congruent ways has been well-documented [25], [50]. However, the relatively thick articular cartilages of archosaurs must have imposed a different loading regime on the subchondral bone, and this is demonstrated by our results showing a very different pattern from mammals.

What would be the advantage of retaining thick articular cartilage at large size? There are certainly disadvantages arising from thick articular cartilage, including the increased likelihood of “ploughing” and shearing [56]. One advantage of thick articular cartilage is shock absorption, a role often suggested for the joints of the giant sauropod dinosaurs [25], [60]. Moreover, in combination with synovial joint fluid, articular cartilage of any thickness serves primarily to reduce joint friction and transmit stress to the subchondral bone [57]. However, we suggest that a major difference between possessing thin or thick articular cartilage is the upper limit at which reduction of stress transmission to the subchondral bone becomes untenable. For example, there may be an upper limit to size associated with thin articular cartilage because a joint can only become so congruent before it can no longer effectively distribute stress to the subchondral bone. Moreover, at some point increasing joint congruence would necessitate thinning an already slim articular cartilage. Thus, eventually a limit will be reached where the joint cannot become more congruent and the articular cartilage cannot become thinner without pathological effects. In fact, the contact area of the articular cartilage demonstrably increases in very large mammals like elephants [58]. In contrast, thick, cartilaginous joints appear to rely on the deformation of thick articular cartilage to diffuse stress [57], and simply expanding the relative size of the joint with increasing size provides an ever larger region over which such deformation can take place (Figure 7). By deforming, any focused regions of stress are blunted [57] and a more diffuse pattern of stress would be transferred to the subchondral bone. In essence, in addition to shock absorption, the ability of thick articular cartilage to deform at increasing scales may aid safe locomotor functions under tremendous weight.

We speculate that these differences in articular cartilage thickness are related to aspects of joint deceleration when the limb is planted on the ground during locomotion. In mammals, highly congruent joints with thin articular cartilage ensure that the bones involved are already in close contact, diminishing the force of deceleration (Figure 7). In contrast, in archosaurs, the close association of the joint surfaces in combination with thick, deformable cartilage would perhaps aid in diminishing joint stress over a much larger range of body sizes (Figure 7). Are there examples of thick deforming cartilaginous tissues enhancing joint function under load? Within the knee of all amniotes are fibrocartilaginous discs known as menisci [61]. In fact, the deformation of menisci plays a critical role in dissipating loads imposed upon the knee joint. For example, when human cadaveric knee joints are loaded after the removal of their menisci, the articular cartilage and subchondral bone experience nearly 120% of the force typically distributed through the joint [62]. Moreover, in kangaroo knees, their relatively supple and broad menisci deform greatly under load, enhancing both joint congruence and safety factors [58]. Although articular cartilage is not fibrocartilage, the menisci demonstrate that, in principle, thick cartilage deformation can effectively reduce stress. Current work by Tsai and Holliday [63] suggests similar mechanisms may be at work in archosaur pelves. Certainly, this is an area of research that deserves further exploration.

Our results also highlight an intriguing postural signal that differs between eutherian mammals and saurischian dinosaurs. The deltopectoral crest of the humerus serves as a region of insertion for the deltoid and pectoral musculature [64]. In quadrupeds, the major actions of the deltopectoral musculature are related to flexion, adduction, medial rotation, and abduction of the humerus at the glenohumeral joint [64], [65]. In our sample, as mammals increase in size from small, sprawling monotremes to giant, columnar-limbed eutherians, the deltopectoral crest migrates from a medially in-turned to a laterally out-turned orientation (Figure 3). In contrast, this landmark becomes markedly medially-oriented with increasing size in our archosaur sample (Figure 5). These differences in deltopectoral crest orientation suggest differences in the major lines of action for the musculature associated with this landmark. What is perhaps most surprising is that although eutherian mammals and saurischian dinosaurs are both descendants of a non-erect common ancestor, it is only in eutherian mammals that deltopectoral crest orientation changes with posture. In fact, the medial orientation of the deltopectoral crest in saurischian dinosaurs compared with non-erect alligators remains or becomes more distinctive within saurischian dinosaurs. The functional significance of these differences goes beyond the scope of our present study, but does suggest that there are important differences in forelimb locomotor adaptations between eutherian mammals and saurischian dinosaurs. For now, we speculate that differences in joint loading and cartilage thickness may play a role in these trends, and future investigations are planned.

Overall, our results indicate that the subchondral shapes of the humerus and femur scale differently between eutherian mammals and saurischian dinosaurs. These differences in subchondral shape are inferred to be correlated with cartilage thickness and its ability to transfer stress to the subchondral bone. For mammals, the narrowing of the subchondral regions and the development of distinct surfaces with increasing size appear to be correlated with thin articular cartilage and congruent joints. As archosaur size increases, subchondral surfaces expand and become gently convex, trends which strongly suggest the retention of thick articular cartilage. Scaling of subchondral shapes in non-erect taxa with thin (monotreme) and thick (alligator) articular cartilages support these associations. Articular cartilage deformation, or lack thereof, may have contributed in significant ways to the attainment and frequency of gigantism in saurischian dinosaurs and eutherian mammals.

Many questions remain, chief among them being what factors would allow the articular cartilage of the largest terrestrial vertebrates to remain viable but pliable. Thick articular cartilage in other reptiles such as leatherback sea turtles (*Dermochelys coriacea*) is heavily invested with cartilage canals and a blood supply [66], characteristics that may have been present in saurischian dinosaurs [25]. Could such cartilaginous “infrastructure” alter the Young’s modulus and deformability of articular cartilage? Moreover, could the cartilages that capped the ends of saurischian dinosaur long bones have been composed both of articular cartilage and fibrocartilage as documented in the tibial plateau of kangaroos [58]? Again, recent work by Tsai and Holliday [63] on archosaur pelvic soft tissues certainly suggest this as a possibility. Future work on such questions may provide clearer insights into the properties of thick articular cartilage.

It would be far too simplistic to suggest that thick articular cartilage was the primary factor driving saurischian dinosaur gigantism. Certainly, a concatenation of biological and environmental variables created a complex mosaic of interactions that contributed to the frequent evolutionary attainment of large body size in non-avian dinosaurs. However, our data suggest that the role of the appendicular skeleton and its soft tissues in promoting gigantism must be examined more thoroughly and beyond simple metrics in conjunction with other factors. Ultimately, a renewed focus on articular cartilage deformation, joint congruence, and the contribution of related tissues such as the fibrocartilaginous menisci of the knee is necessary and warranted. Most certainly, future investigations into how these soft tissues respond to the dynamic demands of locomotion and weight support promise to yield intriguing insights regarding body size in amniotes large and small.

## Supporting Information

Table S1 Mammal specimens utilized in the study.
(DOCX)

Table S2 Archosaur specimens utilized in the study.
(DOCX)
