# Supplementary material for: What Lies Beneath: Sub-Articular Long Bone Shape Scaling in Eutherian Mammals and Saurischian Dinosaurs Suggests Different Locomotor Adaptations for Gigantism
Source: PLoS One. 2013 Oct 9;8(10):e75216. doi: 10.1371/journal.pone.0075216 (PMC3793987; doi:10.1371/journal.pone.0075216)
Supplement: Table S1 — Mammal specimens utilized in the study. (DOCX) [file pone.0075216.s001.docx]

Table S1. Mammal specimens utilized in the study. Specimens are identified by genus only. Eutheria was divided into several clades: Afrotheria, Perissodactyla (rhinos and tapirs only), and Felidae. Institutional abbreviations as per the main text. *, Data taken from the literature.

| Clade | Taxon | Humerus | Femur |
| --- | --- | --- | --- |
| Perissodactyla | *Aphelops* | AMNH 114800 | AMNH 1148038 |
|  |  | AMNH 114799 | AMNH Uncat |
|  | *Ceratotherium* | FMNH 29174 | FMNH 29174 |
|  | *Diceros* | FMNH 121646 | FMNH 121646 |
|  |  | FMNH 57809 | FMNH 57809 |
|  |  | FMNH 127848 | FMNH 127848 |
|  |  | FMNH 127849 | FMNH 127849 |
|  |  | FMNH 60784 |  |
|  | *Indricotherium* | AMNH 26191 | AMNH 21619 |
|  |  |  | AMNH 26393 |
|  | *Menoceras* | AMNH 14214 | AMNH Uncat1 |
|  |  | AMNH 22487 | AMNH Uncat2 |
|  |  | AMNH uncat#8 | AMNH Uncat3 |
|  |  | AMNH uncat2 |  |
|  | *Peraceras* | AMNH 114970 | AMNH 114970 |
|  |  | AMNH 114954 |  |
|  | *Rhinoceros* | FMMH 29124 | FMNH 29124 |
|  |  | FMNH 57639 | FMNH 57639 |
|  |  | FMNH 57822 | FMNH 57822 |
|  | *Tapiris* | FMNH 134536 | FMNH 134536 |
|  |  | FMNH 53937 | FMNH 53937 |
|  |  | FMNH 60010 | FMNH 60010 |
|  |  | FMNH 60768 | FMNH 60768 |
|  |  | AMNH 114588 | AMNH 114588 |
|  |  | AMNH uncatFla.227-3424 | AMNH 115243 |
|  | *Teloceras* | AMNH 16386 | AMNH 115357 |
|  |  | AMNH 115337 | AMNH 115368 |
|  |  | AMNH uncatFla.41-673 | AMNH uncatBX4#7 GroverCol 1931 |
|  |  | AMNH uncatFla.213.3201 | AMNH uncatFla 124-2202 |
|  |  |  | AMNH uncatFla 124-2203 |
|  |  |  | AMNH uncatFla 251-6829 |
| Afrotheria | *Deinotherium* [1] | SU Pl 312/8 | SU Pl 312/8 |
|  | *Dendrohyrax* | AMNH 187789 | AMNH 187789 |
|  |  | AMNH 187794 | AMNH 187794 |
|  |  | AMNH 187790 | AMNH 187790 |
|  |  | AMNH 88428 | AMNH 88428 |
|  |  | AMNH 52120 | AMNH 52120 |
|  | *Elephas* | FMNH 60601 | FMNH 60601 |
|  |  | FMNH 49894 | AMNH 42496 |
|  |  | AMNH 54452 | AMNH 39085 |
|  |  | AMNH 39085 | AMNH 39081 |
|  |  | AMNH 39081 | AMNH 39088 |
|  |  | AMNH 39082 |  |
|  | *Heterohyrax* | AMNH 54007 | AMNH 54007 |
|  |  | AMNH 187797 | AMNH 187797 |
|  |  | AMNH 187796 | AMNH 187796 |
|  |  | FMNH 18842 | FMNH 18842 |
|  | *Loxodonta* | AMNH 88402 | AMNH 88402 |
|  |  | AMNH 88404 | AMNH 88404 |
|  |  | AMNH 88403 | AMNH 88403 |
|  |  | AMNH 52093 | AMNH 52093 |
|  |  | AMNH 90176 | AMNH 90176 |
|  | *Mammut* | FMNH 67BS71 | FMNH 67BS71 |
|  |  | FMNH 401BS71 | FMNH 401BS71 |
|  |  | FMNH 533BS71 | FMNH 533BS71 |
|  |  | FMNH 548BS71 | FMNH 548BS71 |
|  |  | FMNH P12803 | FMNH P12803 |
|  |  | FMNH P12804 | FMNH P12804 |
|  |  | FMNH P14305 | FMNH P14305 |
|  |  | FMNH PM3945 | FMNH PM3945 |
|  |  | FMNH PM39394 | FMNH PM39394 |
|  |  | FMNH XXXBS71b | FMNH XXXBS71b |
|  | *Orycteropus* | FMNH 99431 | FMNH 99431 |
|  |  | FMNH 57913 | FMNH 57913 |
|  |  | FMNH 161408 | FMNH 161408 |
|  | *Procavia* | AMNH 202324 | AMNH 202324 |
|  |  | AMNH 35326 | AMNH 35326 |
| Felidae | *Acinonyx* | FMNH 34589 | FMNH 34589 |
|  |  | FMNH 29633 | FMNH 29633 |
|  |  | FMNH 57826 | FMNH 57826 |
|  |  |  | FMNH 60060 |
|  | *Caracal* | FMNH 95922 | FMNH 95922 |
|  |  | FMNH 57220 | FMNH 57220 |
|  | *Felis* | FMNH 99026 | FMNH 97867 |
|  |  | FMNH 97867 | FMNH 125115 |
|  |  | FMNH 104412 | FMNH 60521 |
|  |  | FMNH 125115 | FMNH 85214 |
|  |  | FMNH 60521 | FMNH 127835 |
|  |  | FMNH 85214 | FMNH 60613 |
|  |  | FMNH 127835 |  |
|  |  | FMNH 60613 |  |
|  | *Leopardus* | FMNH 52488 | FMNH 121293 |
|  |  | FMNH 134485 | FMNH 159997 |
|  |  | FMNH 68895 | FMNH 68895 |
|  |  | FMNH 121293 | FMNH 52488 |
|  |  | FMNH 159997 | FMNH 134485 |
|  | *Lynx* | FMNH 195429 | FMNH 195429 |
|  |  | FMNH 195437 | FMNH 195437 |
|  |  | FMNH 129344 | FMNH 129344 |
|  | *Neofelis* | FMNH 186436 | FMNH 186436 |
|  |  | FMNH 104730 | FMNH 104730 |
|  |  | FMNH 183653 | FMNH 183653 |
|  |  |  | FMNH 54304 |
|  | *Panthera* | FMNH 15530 | FMNH 60788 |
|  |  | FMNH 60788 | FMNH 135270 |
|  |  | FMNH 135270 | FMNH 49340 |
|  |  | FMNH 49340 | FMNH 54639 |
|  |  | FMNH 54639 | FMNH 173259 |
|  |  | FMNH 173259 | FMNH 159998 |
|  |  | FMNH 159998 | FMNH 54321 |
|  |  | FMNH 54321 | FMNH 57177 |
|  |  | FMNH 57177 | FMNH 60626 |
|  |  | FMNH 60626 | FMNH 127842 |
|  |  | FMNH 127842 | FMNH 153777 |
|  |  | FMNH 153777 | FMNH 188486 |
|  |  | FMNH 159999 | FMNH 60760 |
|  |  | FMNH 188486 | FMNH 57172 |
|  |  | FMNH 60760 |  |
|  |  | FMNH 57172 |  |
|  | *Pardofelis* | FMNH 104901 | FMNH 104901 |
|  |  | FMNH 60358 | FMNH 60358 |
|  |  | FMNH 68728 | FMNH 68728 |
|  |  | FMNH 60020 | FMNH 60020 |
|  | *Prionailurus* | FMNH 114386 | FMNH 114386 |
|  |  | FMNH 62896 | FMNH 62896 |
|  |  | FMNH 168869 | FMNH 168869 |
|  | *Profelis* | FMNH 99025 | FMNH 99025 |
|  |  | FMNH 121528 | FMNH 121528 |
|  | *Puma* | FMNH 51472 | FMNH 51472 |
|  |  | FMNH 129339 | FMNH 129339 |
|  |  | FMNH 30818 | FMNH 30818 |
|  | *Uncia* | FMNH 160000 | FMNH 160000 |
|  |  | FMNH 127297 | FMNH 127297 |
|  |  | FMNH 104940 |  |
| Monotremata | *Ornithorhynchus* | FMNH 160034 | FMNH 160034 |
|  |  | FMNH 60899 | FMNH 60899 |
|  |  | FMNH 64346 | FMNH 64346 |
|  |  | FMNH 60902 | FMNH 60902 |
|  |  | FMNH 160036 | FMNH 160036 |
|  |  | FMNH 160035 | FMNH 160035 |
|  |  | FMNH 160033 | FMNH 160033 |
|  |  | FMNH 160038 | FMNH 160038 |
|  | *Tachyglossus* | FMNH 60905 | FMNH 60905 |
|  |  | FMNH 57291 | FMNH 57291 |
|  |  | FMNH 60903 | FMNH 60903 |
|  |  | FMNH 57804 | FMNH 57804 |
|  |  | FMNH 77042 | FMNH 77042 |

**References**

1. Kovachev D, Nikolov I (2006) *Deinotherium thraceiensis* sp. nov. from the Miocene near Ezerovov, Plovdiv District. Geol Balc 35: 5–40.
