# Supplementary material for: What Lies Beneath: Sub-Articular Long Bone Shape Scaling in Eutherian Mammals and Saurischian Dinosaurs Suggests Different Locomotor Adaptations for Gigantism
Source: PLoS One. 2013 Oct 9;8(10):e75216. doi: 10.1371/journal.pone.0075216 (PMC3793987; doi:10.1371/journal.pone.0075216)
Supplement: Table S2 — Archosaur specimens utilized in the study. (DOCX) [file pone.0075216.s002.docx]

Table S2. Archosaur specimens utilized in the study. Specimens are identified by genus only. Saurischia was divided into Sauropodomorpha and Theropoda (Aves inclusive). Institutional abbreviations as per the main text. *, Data taken from the literature.

| Clade | Taxon | Humerus | Femur |
| --- | --- | --- | --- |
| Sauropodomorpha | *Antetonitrus* | BP/1/4952 | BP/1/4952 |
|  | *Apatosaurus* | AMNH 6114 | BYU Display |
|  |  | BYU Display | MWC Display |
|  |  | CMNH 21715 | OMNH 01991 |
|  |  | CMNH 3018 |  |
|  |  | DNM 2943 |  |
|  |  | DNM 3923 |  |
|  |  | MWC 645 |  |
|  |  | YPM 1980 |  |
|  | *Barosaurus lentus* | AMNH 6341 | AMNH 6341 |
|  | *Torneria ("Barosaurus")* | MBR.2637 | MBR.2637 |
|  |  | MBR.2660 | MBR.2660 |
|  |  | MBR.2665 | MBR.2665 |
|  |  | MBR.2670 | MBR.2670 |
|  |  | MBR.2700 | MBR.2700 |
|  | *Brachiosaurus* | MBR.2096-1 | MBR.2096-1 |
|  |  | MBR.2694.1 | MBR.2694.1 |
|  |  | MBR.2699 | MBR.2699 |
|  | *Camarasaurus* | YPM MOUNT | AMNH 435 |
|  |  | AMNH 435 | BYU 725/13643 |
|  |  | BYU 725/13643 | MWC Display |
|  |  | MWC Display | OMNH 01794 |
|  |  | UMNH 5286 | UMNH 5286 |
|  |  | OMNH 01794 | YPM 5862 |
|  |  |  | YPM MOUNT |
|  | *Diplodocus* | AMNH 5855 | AMNH 5855 |
|  |  | BYU 725/4889 | BYU 725/4889 |
|  |  | CM 21788 | CM 21788 |
|  |  | CM 30726 | CM 30726 |
|  |  | OMNH 01793 | OMNH 01793 |
|  |  | UMNH 82 | UMNH 82 |
|  | *Epachthosaurus** | UNPSJB-PV920 [1] | UNPSJB-PV920 [1] |
|  | *Nequenasaurus** | MLPCS 1050 [2] | MLPCS 1050 [2] |
|  |  | MLPCS 1099 [3] | MLPCS 1099 [3] |
|  | *Opisthocoelicaudia** | ZPAL MgD-I/48 [4] | ZPAL MgD-I/48 [4] |
|  | *Paralititan** | CGM 81119 [5] | CGM 81119 [5] |
|  | *Rapetosaurus** | FMNH PR 2209 [6] | FMNH PR 2209 [6] |
|  | *Saltasaurus** | PVL 4017-79 [3] | PVL 4017-79 [3] |
| “Prosauropods” | *Euskelosaurus* | BP/1/5256 | BP/1/5256 |
|  |  | SAM-PK-K 3602 | SAM-PK-K 3602 |
|  |  | SAM-PK-K 3603 | SAM-PK-K 3603 |
|  | *Melanorosaurus* | BP/1/4266 | SAM 3450 |
|  | *Massospondylus* | BP/1/4693 | BPI/1/4267 |
|  |  | BP/1/4769 | BP/1/4693 |
|  |  | BP/1/4777 | SAM-PK-K 7896 |
|  |  | BP/1/4786 | SAM-PK-K xx (JUVE) |
|  |  | BP/1/4861 | BP/1/4786 |
|  |  | BP/1/4953 | BP/1/4953 |
|  |  | BP/1/4998 | BP/1/4777 |
|  |  | BPI/1/4267 | BP/1/4861 |
|  |  | SAM 2772 | BP/1/4998 |
|  |  | SAM 3450 | SAM 5135 |
|  |  | SAM 5135 | BP/1/4266 |
|  |  | SAM-PK-K |  |
|  |  | SAM-PK-K 7896 | BP/1/4769 |
|  | *Plateosaurus* | SMNS 13200 | SMNS 13200 |
|  |  | SMNS 1967108 | SMNS 1967108 |
|  |  | SMNS 53537 | SMNS 53537 |
|  |  | SMNS F27 | SMNS F27 |
|  |  | SMNS F48 | SMNS F48 |
|  |  | SMNS Tross1932 | SMNS Tross1932 |
| Theropoda | *Allosaurus* | UMNHVP 12231 | UMNHVP 12231 |
|  |  | UMNHVP 1226B | UMNHVP 1226B |
|  |  | UMNHVP 3208 | UMNHVP 3208 |
|  |  | UMNHVP 5409 | UMNHVP 5409 |
|  |  | UMNHVP 6317 | UMNHVP 6317 |
|  |  | UMNHVP 7149 | UMNHVP 7149 |
|  |  | UMNHVP 7882 | UMNHVP 7882 |
|  |  | UMNHVP 7884 | UMNHVP 7884 |
|  |  | UMNHVP 7885 | UMNHVP 7885 |
|  |  | UMNHVP 7886 | UMNHVP 7886 |
|  |  | UMNHVP 7891 | UMNHVP 7891 |
|  |  | UMNHVP 7892 | UMNHVP 7892 |
|  |  | UMNHVP 7893 | UMNHVP 7893 |
|  |  | UMNHVP 7894 | UMNHVP 7894 |
|  |  | UMNHVP 7895 | UMNHVP 7895 |
|  |  | UMNHVP 7905 | UMNHVP 7905 |
|  |  | UMNHVP 7906 | UMNHVP 7906 |
|  |  | UMNHVP 7908 | UMNHVP 7908 |
|  |  | UMNHVP 7909 | UMNHVP 7909 |
|  |  | UMNHVP 7910 | UMNHVP 7910 |
|  |  | UMNHVP 9480 | UMNHVP 9480 |
|  | *Numida* | WIU uncataloged specimen 1 | WIU uncataloged specimen 1 |
|  |  | WIU uncataloged specimen 2 | WIU uncataloged specimen 2 |
|  |  | WIU uncataloged specimen 3 | WIU uncataloged specimen 3 |
|  |  | WIU uncataloged specimen 4 | WIU uncataloged specimen 4 |
|  |  | WIU uncataloged specimen 5 | WIU uncataloged specimen 5 |
|  |  | WIU uncataloged specimen 6 | WIU uncataloged specimen 6 |
|  |  | WIU uncataloged specimen 7 | WIU uncataloged specimen 7 |
|  |  | WIU uncataloged specimen 8 | WIU uncataloged specimen 8 |
|  |  | WIU uncataloged specimen 9 | WIU uncataloged specimen 9 |
|  |  | WIU uncataloged specimen 10 | WIU uncataloged specimen 10 |
| Crocodylia | *Alligator* | WIU 1CF | WIU 1CF |
|  |  | WIU 49.5 inch | WIU 49.5 inch |
|  |  | WIU 52 inch | WIU 52 inch |
|  |  | WIU 52 inch female (#2) | WIU 52 inch female (#2) |
|  |  | WIU 52.5 inch X | WIU 52.5 inch X |
|  |  | WIU 54.5 inch 908156 | WIU 54.5 inch 908156 |
|  |  | WIU 61 inch | WIU 61 inch |
|  |  | WIU 72 inch | WIU 72 inch |
|  |  | WIU 84 inch | WIU 84 inch |
|  |  | WIU DS-10 | WIU DS-10 |
|  |  | WIU DS-7 | WIU DS-7 |
|  |  | WIU X | WIU X |

**References**

1. Martinez RD, Gimenez O, Rodriguez J, Luna M, Lamanna MC (2004) An articulated specimen of the basal Titanosaurian (Dinosauria: Sauropoda) *Epachthosaurus scuiottoi* from the Early Late Cretaceous Bajo Barreal Formation of Chubut Province, Argentina. J Vertebr Paleontol 24: 107–120.

2. Otero A (2010) The Appendicular Skeleton of *Neuquensaurus* , a Late Cretaceous Saltasaurine Sauropod from Patagonia, Argentina. Acta Palaeontol Pol 55: 399–426. doi:10.4202/app.2009.0099.

3. Powell JE (2003) Revision of South American Titanosaurid dinosaurs: palaeobiological, palaeobiogeographical, and phylogenetic aspects. Rec Queen Vic Mus Launceston 111: 1–174.

4. Borsuk-Bialynicka M (1977) A new Camarasaurid sauropod *Opisthocoelicaudia skarzynskii* gen. n., sp. n. from the Upper Cretaceous of Mongolia. Palaeontol Pol 37: 5–64.

5. Smith JB, Lamanna MC, Lacovara KJ, Dodson P, Smith JR, et al. (2001) A Giant Sauropod Dinosaur from an Upper Cretaceous Mangrove Deposit in Egypt. Science 292: 1704–1706. doi:10.1126/science.1060561.

6. Rogers KC (2009) The postcranial osteology of *Rapetosaurus krausei* (Sauropoda: Titanosauria) from the Late Cretaceous of Madagascar. J Vertebr Paleontol 29: 1046–1086. doi:10.1671/039.029.0432.
